# Autoimmune polyglandular syndrome type 2 in pregnancy: a case report and review of the literature

**DOI:** 10.1515/crpm-2020-0058

**Published:** 2022-02-23

**Authors:** David R. Bayless, Trevor M. Caldarera, Hassan M. Harirah

**Affiliations:** Department of Obstetrics and Gynecology, Division of Maternal-Fetal Medicine, University of Texas Medical Branch, Galveston, TX, USA

**Keywords:** autoimmune polyglandular syndrome type-2, endocrinopathies; preeclampsia with severe features; pregnancy

## Abstract

**Objectives:**

Autoimmune polyglandular syndromes are uncommon heterogeneous conditions characterized by the association of two or more organ-specific endocrinopathies. Very few cases of these syndromes have been described during pregnancy. Here we report a case of autoimmune polyglandular syndrome type-2 presenting during pregnancy and complicated by preeclampsia with severe features.

**Case presentation:**

The patient is a 35-year-old G7P0242 woman with a history of adrenal insufficiency, type 1 diabetes mellitus, and chronic lymphocytic thyroiditis. She was admitted to our institution at 34 weeks’ gestation for preterm contractions, nausea, and lower extremity edema for a few weeks prior to admission. At 35 weeks’ gestation, she developed preeclampsia with severe features requiring repeat cesarean section with good maternal and fetal outcomes. Recognizing the occurrence of this rare syndrome during pregnancy, adequate replacement of the deficient hormones, and close maternal and fetal surveillance are essential to achieving favorable outcomes.

**Conclusions:**

To our knowledge, this is the first reported case of a pregnant woman with autoimmune polyglandular syndrome type-2 complicated by preeclampsia with severe features.

## Introduction

Autoimmune polyglandular syndromes (APSs) are a rare inherited array of disorders characterized by multiple endocrine end-organ dysfunction. They are classified into three types based on clinical presentation. Types 1 and 2 are well-defined entities with type-3 being ill defined. The autoimmune nature of these disorders is based on the presence of lymphocytic infiltration of the affected glands, organ-specific autoantibodies in the serum, and an association with HLA linkage genes [1].

Autoimmune polyglandular syndrome type 2 (APS-2) is a relatively uncommon autoimmune condition defined as the combination of primary adrenal insufficiency (Addison’s disease), autoimmune thyroid disease (AITD), and/or type 1 diabetes mellitus. APS-2 has a prevalence of approximately five per 100,000 in the United States and is most commonly diagnosed in middle-aged women [1]. The clinical presentation of APS-2 shows great heterogeneity and the condition often manifests sequentially with a large time interval between the occurrence of the first and second endocrine diseases. Patients with APS-2 are also predisposed to additional autoimmune diseases such as vitiligo, hypergonadotropic hypogonadism, and autoimmune atrophic gastritis [1].

Since both thyroid and adrenal gland insufficiency can be individually associated with decreased fertility, the presentation of APS-2 is rarely encountered during pregnancy. Additionally, pregnant women with individual endocrine disorders are more prone to maternal and fetal complications, including miscarriage, preterm birth, fetal growth restriction, and preeclampsia [2], [3], [4]. Therefore, it is important to recognize this rare syndrome during pregnancy for its potentially unfavorable outcomes. Here, we present an interesting case of APS-2 presenting during pregnancy complicated by preeclampsia with severe features. She delivered at 35 weeks’ gestation with favorable outcomes for both mother and baby.

## Case presentation

A 35-year-old Caucasian G7P0242 female at 34 weeks and five days’ gestation by ultrasound presented to Labor and Delivery of our institution with a one-day history of preterm contractions. She reported nausea and lower extremity edema of a few weeks’ duration before her presentation. She denied vaginal bleeding, leakage of fluid, or decreased fetal movement. She had undergone a fetal anatomy ultrasound 12 days prior to presentation that showed no obvious malformations and an estimated fetal weight of 2,702 g (95th percentile).

The patient’s obstetric history was remarkable for four spontaneous abortions that each occurred prior to 20 weeks’ gestation with no workup for underlying causes pursued. She had had two preterm deliveries via cesarean section at 31 and 34 weeks’ gestation, respectively. The patient did not recall why she delivered prematurely; she only remembered that she had “very low glucose” prior to both deliveries. She had only a few sporadic prenatal visits notable for uncontrolled diabetes and hypothyroidism.

Patient’s past medical history was significant for type 1 diabetes mellitus treated with insulin lispro administered via insulin pump (total of 34 units daily), primary adrenal insufficiency treated with prednisone 10 mg and fludrocortisone 0.1 mg daily, and chronic lymphocytic thyroiditis treated with levothyroxine 225 mcg daily. These conditions were diagnosed around the age of five years. Review of her medical records revealed positive testing for thyroxine peroxidase (TPO) antibodies but negative for 21-hydroxylase antibodies. An ACTH stimulation test had been scheduled but the patient did not show up for her appointment. Significantly, the patient had a history of medication noncompliance and had been hospitalized approximately 20–25 times for diabetic ketoacidosis since adolescence, with at least one hospitalization for adrenal crisis as well. Her hemoglobin A1c and thyroid-stimulating hormone (TSH) one month prior to the current presentation were elevated at 9.7% and 276 mIU/L, respectively. She denied a family history of autoimmune disease and denied smoking, illicit drug use, and regular consumption of alcohol.

On physical exam, the patient was afebrile, pulse was 70/min, and blood pressure ranged from 140 to 150/70 mmHg. She appeared pallid but said she had “always been this way.” Her cardiac and respiratory exams were unremarkable and she did not have any mucosal or cutaneous hyper- or hypopigmentation. On pelvic exam, the cervix was not dilated with fetal cephalic presentation noted. External tocometer showed uterine contractions every 2–3 min with reactive fetal heart tracing. Initial laboratory tests revealed microcytic anemia with hemoglobin of 6.7 g/dL, mean corpuscular volume (MCV) 77.3 fL and decreased iron saturation of 8%. The urine protein/creatinine ratio was elevated at 3.3 with a serum creatinine of 0.84 mg/dL, BUN of 12 mg/dL, and LDH of 484 U/L. Based on her past medical history and presentation, patient was diagnosed with APS-2 and admitted to the antepartum service for management of preterm contractions, microcytic anemia, and preeclampsia without severe features.

During the first three days of hospitalization, patient’s blood pressure remained around 130/80 mmHg without medication. She was maintained on her prenatal dose of insulin lispro, levothyroxine, prednisone, and fludrocortisone. Her blood glucose levels ranged from 85 to 200 mg/dL. With IV hydration and rest preterm contractions subsequently resolved. On the fourth day, pitting edema of lower extremities was noted. By the evening of the fifth day, the edema extended from her feet up to her waist and the patient developed shortness of breath. Her blood pressure was noted to be 161/66 mmHg, serum creatinine had risen to 1.08 mg/dL, and serum lactate dehydrogenase (LDH) increased to 652 U/L. Based on these clinical and laboratory changes, the diagnosis of preeclampsia with severe features was established. Given the patient’s worsening symptoms and laboratory results, the decision was made to proceed with delivery. She received 5 mg IV hydralazine for hypertension control and a 2 g/h infusion of IV magnesium sulfate for seizure prophylaxis. Due to her history of two prior cesarean deliveries and patient’s request for permanent sterilization, a repeat cesarean section and bilateral tubal ligation was performed at 35 weeks’ gestation. Intraoperative estimated blood loss was 600 mL. Since her preoperative hemoglobin of 6.6 g/dL, patient received intraoperatively 2 units of packed red blood cells (pRBCs), 20 U of IV oxytocin, 250 mcg of IM carboprost, and 10 U of intrauterine oxytocin to control blood loss. A male infant was delivered with Apgar scores of 7 and 9 at 5 and 10 min, respectively. The newborn developed moderate respiratory distress soon after delivery and was transferred to the neonatal intensive care unit (NICU) for respiratory support.

The patient’s postoperative course was complicated by persistent elevation of blood pressure ranging from 150/80 to 170/90 mmHg, hyperglycemia, and pulmonary edema. The pulmonary edema was adequately treated with furosemide 20 mg IV, and hypertension managed with oral labetalol 100 mg twice daily. The postoperative hyperglycemia was controlled with IV regular insulin drip. The patient was discharged home four days after delivery after achieving adequate control of her blood pressure and serum glucose. The infant was discharged in good condition one week after delivery. At her two weeks’ postoperative visit, the patient had an uneventful recovery with good wound healing.

## Discussion

The presented case is for a patient with APS-2 who was admitted at 34 weeks’ gestation with preeclampsia. Workup confirmed uncontrolled type 1 diabetes mellitus and hypothyroidism. During admission, the symptoms and signs of preeclampsia progressed rapidly to severe features that required delivery via repeat cesarean section at 35 weeks with favorable maternal and fetal outcomes.

APSs comprise a diverse group of clinical conditions characterized by functional impairment of multiple endocrine glands. They are characterized by involvement of at least two organ-specific endocrine autoimmune disorders, insidious presentation, and circulating autoantibodies and lymphocytic infiltration of the affected organs. There is marked variation in the frequencies and APSs often coexist with other autoimmune conditions that affect non-endocrine organs [1, 5]. The two most important forms of APS are the juvenile and adult types. The juvenile type (APS-1) is caused by mutations in the autoimmune regulator (AIRE) gene on chromosome 21, and is defined by the combination of chronic mucocutaneous candidiasis, Addison’s disease, and hypoparathyroidism. The adult type (APS-2) is a multigenetic disorder associated with a number of HLA haplotypes [1, 5]. APS-2 is more common than the juvenile type and exhibits the combination of type 1 diabetes mellitus, autoimmune thyroid disease, Addison’s disease, and other autoimmune disorders [5].

APS-2 is the most common of the APSs yet is still a relatively rare condition, with an estimated prevalence of five per 100,000 in the United States [1]. Like other autoimmune conditions, APS-2 is found more commonly in women than in men [5]. Almost all patients with APS-2 have Addison’s disease; around 70–80% have autoimmune thyroiditis; and 30–50% have type 1 diabetes mellitus. Approximately 5–10% develop autoimmune hepatitis, atrophic gastritis with vitamin B12 deficiency, vitiligo, and/or hypergonadotropic hypogonadism [1]. However, APS-2 is typically not diagnosed until middle age, possibly because up to 20 years may elapse between the onset of the first and second endocrinopathies. Similar to the presentation in our patient, APS-2 usually begins to manifest during childhood or adolescence.

Diagnosis of APS-2 is often overlooked because of heterogeneous symptoms and the slowly progressive nature of the individual endocrine deficits. Patients with Addison’s disease classically report symptoms of hypocortisolism and hypoaldosteronism, includingfatigue, weight loss, weakness, nausea, and craving for salt and sugar. Patients with autoimmune lymphocytic thyroiditis report symptoms of hypothyroidism such as fatigue, weight gain, constipation, dysphoria, and cold intolerance. Patients with type 1 diabetes mellitus report symptoms of hypoinsulinemia such as polydipsia, polyuria, and weight loss. These symptoms of APS-2 might be subtle upon initial presentation, with mostly nonspecific complaints such as fatigue and unintentional weight changes. Conversely, the initial presentation may come in the form of life-threatening sequelae of untreated disease such as diabetic ketoacidosis, vasomotor shock with Addison’s disease, and myxedema coma with AITD. In our presented case, the patient had been hospitalized numerous (20–25) times with ketoacidosis episodes and at least once for adrenal crisis.

Recognizing the manifestations of APS-2 in pregnant women can be difficult as there is significant overlap between the physiological changes associated with pregnancy and the nonspecific symptoms of APS-2. Both adrenal insufficiency and normal pregnancy can present with nausea, fatigue, and cutaneous hyperpigmentation, for example. Adding to this difficulty is the fact that most patients with APS-2 begin to develop clinically apparent autoimmune disease in adolescence, and the majority of obstetric patients are usually in their late adolescence or early adulthood. Younger women with APS-2 may begin to develop symptoms of their autoimmune disorder around the time of their first pregnancy. On the other hand, pregnancy is a physiologically immunotolerant state and symptoms of an underlying autoimmune thyroiditis are unlikely to manifest for the first time during pregnancy [6]. Therefore, careful history and physical examination, with judiciously chosen laboratory tests, are crucial to discern the pregnant patient at significant risk for APS-2 from normal pregnant women.

In our patient, diagnosis of her autoimmune conditions was established before her current pregnancy. Nonetheless, she had inadequate prenatal care and was noncompliant in taking her medications. She presented with preterm contraction secondary to dehydration as a result for hyperglycemia and nausea probably due to glucocorticoid under-replacement. Adequate hydration and control of her blood glucose helped in resolution of preterm contractions. She was noted to have microcytic iron deficiency anemia, probably due to her chronic disease and possible malabsorption. In addition to the constellation of APS-2 endocrinopathies, our patient was found to have elevated blood pressure and proteinuria consistent with preeclampsia. Preeclampsia is a disorder of pregnancy characterized by new-onset hypertension accompanied by proteinuria and other signs of severe organ dysfunction in the second half of pregnancy. It is a serious disorder, which can lead to maternal and fetal morbidity and mortality. In our patient’s case, features of severe preeclampsia developed rapidly within one week of her admission. She also had two prior premature deliveries, which we presume were probably related to preeclampsia complications given that she delivered via cesarean sections.

Management of APS-2 in pregnancy consists of treating the particular autoimmune diseases affecting the patient as though each endocrine disorder had occurred separately. For Addison’s disease, the patient should receive glucocorticoid and mineralocorticoid replacement therapy. Hydrocortisone is the preferred glucocorticoid in obstetric patients since it does not cross the placenta [4]. For patients who began adrenal insufficiency treatment prior to becoming pregnant, the dosages of hormone replacement therapy will often need to be adjusted to match the hormonal changes that occur in normal pregnancy [4]. For type 1 diabetes mellitus, the patient will require insulin, the dosage of which may also need to be adjusted during each trimester of the pregnancy. For autoimmune thyroiditis, patients require thyroid hormone replacement, usually with levothyroxine. For most patients, the dosage of levothyroxine will have to be increased by around 30% above pre-pregnancy levels due to increased maternal synthesis of thyroxine-binding globulin (TBG) [7]. Importantly, hypothyroid patients with suspected adrenal insufficiency should not receive thyroid hormone replacement until glucocorticoid administration, as thyroid hormone replacement in the setting of hypocortisolism can precipitate an adrenal crisis [8].

Very few cases of APS-2 have been described during pregnancy. In the first trimester, APS-2 can complicate pregnancy and be confused with hyperemesis gravidarum as a cause of hypoglycemia and electrolyte imbalance. Another interesting case report documents how in the third trimester, a pregnant patient was diagnosed with autoimmune thyroiditis and a history of HELLP (hemolysis, elevated liver enzymes, and low platelets) syndrome in a prior pregnancy [9]. After her levothyroxine dose was increased, she developed adrenal crisis. During normalization of her adrenal function, she then developed diabetes insipidus. The report shows how pregnancy may influence the course of preexisting APS-2 and lead to unmasking of its component endocrine dysfunctions [9].

Preoperative dosing of stress glucocorticoids prior to delivery has long been considered the norm for obstetrical patients who were on long-term glucocorticoid therapy during pregnancy. However, recent evidence suggests that this traditional approach may be unnecessary [10]. In line with this, we opted against administering stress glucocorticoids to our patient. During surgery, intraoperative bleeding in the setting of preexisting anemia was controlled with uterotonics (oxytocin and carboprost) and transfusion of two units of pRBCs. The postoperative course was mostly uneventful and the patient did not experience any evidence of adrenal insufficiency.

To our knowledge, this is the first reported case of APS-2 complicated by preeclampsia with severe features and resulting in favorable maternal and fetal outcomes. Diagnosis of APS-2 should be entertained for obstetric patients with a history of autoimmune disease who develop symptoms and signs of an additional endocrine insufficiency. Recognizing this diagnosis, proper hormone supplementation, and close maternal and fetal surveillance for obstetric complications such as preeclampsia are necessary for an optimal outcome.
